# Novel and recurrent *PITX3* mutations in Belgian families with autosomal dominant congenital cataract and anterior segment dysgenesis have similar phenotypic and functional characteristics

**DOI:** 10.1186/1750-1172-9-26

**Published:** 2014-02-20

**Authors:** Hannah Verdin, Elena A Sorokina, Françoise Meire, Ingele Casteels, Thomy de Ravel, Elena V Semina, Elfride De Baere

**Affiliations:** 1 Center for Medical Genetics, Ghent University and Ghent University Hospital, Ghent, Belgium; 2 Department of Pediatrics and Children’s Research Institute, Medical College of Wisconsin, Milwaukee 53226, WI, USA; 3 Department of Paediatric Ophthalmology, Queen Fabiola Children’s University Hospital, Brussels, Belgium; 4 Department of Ophthalmology, Leuven University Hospitals, Leuven, Belgium; 5 Center for Human Genetics, Leuven University Hospitals, Leuven, Belgium; 6 Department of Cell Biology, Neurobiology and Anatomy, Medical College of Wisconsin, Milwaukee 53226, WI, USA

## Abstract

**Background:**

Congenital cataracts are clinically and genetically heterogeneous with more than 45 known loci and 38 identified genes. They can occur as isolated defects or in association with anterior segment developmental anomalies. One of the disease genes for congenital cataract with or without anterior segment dysgenesis (ASD) is *PITX3*, encoding a transcription factor with a crucial role in lens and anterior segment development. Only five unique *PITX3* mutations have been described, of which the 17-bp duplication c.640_656dup, p.(Gly220Profs*95), is the most common one and the only one known to cause cataract with ASD. The aim of this study was to perform a genetic study of the *PITX3* gene in five probands with autosomal dominant congenital cataract (ADCC) and ASD, to compare their clinical presentations to previously reported *PITX3*-associated phenotypes and to functionally evaluate the *PITX3* mutations found.

**Methods:**

Sanger sequencing of the coding region and targeted exons of *PITX3* was performed in probands and family members respectively. Transactivation, DNA-binding and subcellular localization assays were performed for the *PITX3* mutations found. Ophthalmological examinations included visual acuity measurement, slit-lamp biomicroscopy, tonometry and fundoscopy.

**Results:**

In four Belgian families with ADCC and ASD the recurrent 17-bp duplication c.640_656dup, p.(Gly220Profs*95), was found in a heterozygous state. A novel *PITX3* mutation c.573del, p.(Ser192Alafs*117), was identified in heterozygous state in a Belgo-Romanian family with a similar phenotype. Functional assays showed that this novel mutation retains its nuclear localization but results in decreased DNA-binding and transactivation activity, similar to the recurrent duplication.

**Conclusions:**

Our study identified a second *PITX3* mutation leading to congenital cataract with ASD. The similarity in phenotypic expression was substantiated by our *in vitro* functional studies which demonstrated comparable molecular consequences for the novel p.(Ser192Alafs*117) and the recurrent p.(Gly220Profs*95) mutations.

## Background

Cataract is defined as opacity or cloudiness of the crystalline lens. It is the primary cause of blindness worldwide and is classified into different types based on the age of onset. Congenital cataract manifests in the first year of life; the estimated incidence of congenital cataract is 72 per 100,000 children in developed countries with a higher incidence in less-developed countries. Congenital cataract is clinically and genetically heterogeneous with about 45 loci known and 38 genes identified. Mutations in genes encoding proteins important in the development and maintenance of the structural integrity of the lens such as crystallins, connexins and aquaporins [1,2] are typically associated with isolated congenital cataracts while mutations in the transcription factor genes *PAX6*[3], *FOXE3*[4], *EYA1*[5], *MAF*[6], and *PITX3*[7] have been described in congenital cataract with anterior segment dysgenesis (ASD). ASD is an umbrella term for the spectrum of developmental disorders affecting the structures of the anterior segment of the eye. The ocular anomalies typically include corneal opacity, adhesions between the iris and cornea or lens and cornea, iris hypoplasia, corectopia or polycoria, and malformation of the irido-corneal angle drainage structures [8].

*PITX3* was isolated as the third gene of the *PITX* homeobox-containing transcription factor gene family [9,10], along with *PITX1*[11,12] and *PITX2*[13]. The PITX protein family is a subfamily of the paired-like class of the homeobox-containing proteins, which play a crucial role in the development of different organisms including mammals. Like the other members of this family, PITX3 contains a characteristic and strongly conserved homeodomain required for DNA binding. The conserved 14-amino acid OAR motif, named after the homeodomain proteins *otp*, *aristaless*, and *rax*[14], is located downstream of this homeodomain, and may function in the target specificity and transactivation of the homeodomain protein [13,14]. Interestingly, *Pitx3* mapped to the *aphakia* (*ak*) locus [9], a recessive mutation resulting in bilateral microphthalmia with lens aplasia originally described by Varnum and Stevens in 1968. The lens develops normally in *ak* mice until an arrest occurs around embryonic days 10.5–11 [15] corresponding to the moment of initial expression of *Pitx3* in the lens [7,9]. Consequently, *Pitx3* was the top candidate for the *ak* phenotype but no mutation was found in the coding region of *Pitx3*[9]. Two different deletions in the promoter region of *Pitx3* were later found to explain the *ak* phenotype [16,17]. Before this finding, the first human *PITX3* mutations had already been identified in two families with autosomal dominant congenital cataract (ADCC). A 17-bp duplication c.640_656dup, p.(Gly220Profs*95), was found in a family with ADCC and ASD while a missense mutation c.38G > A, (Ser13Asn), was identified in a second family [7]. Since the original gene identification study, there have been only a few additional mutations identified, bringing the total number of unique *PITX3* mutations found in ADCC with or without ASD to five mutations in 13 different families [7,18-26]. Two of these mutations are recurrent, the most common one being the 17-bp duplication p.(Gly220Profs*95) which was reported in eight of the 13 families [7,18-21,23,24]. The other recurrent mutation is a 1-bp deletion at position 650, p.(Gly217Alafs*92), found in two families [18,22]. Thus far, only the 17-bp duplication has been associated with ASD; all other mutations are reported with isolated cataract. Of particular note, homozygous *PITX3* mutations were also described in two consanguineous pedigrees [22,25].

The aim of this study was to analyze the *PITX3* gene in five Belgian families with ADCC and ASD. We identified the recurrent 17-bp duplication c.640_656dup, p.(Gly220Profs*95), in four of these families, and a novel *PITX3* mutation c.573del, p.(Ser192Alafs*117), in a fifth family. *In vitro* functional assays were performed for both mutations, showing similar functional characteristics. In conclusion, the similar ADCC and ASD phenotypes resulting from both mutations could be explained by our *in vitro* functional studies.

## Methods

### Patients

The consenting families enrolled in this study were referred for clinical genetic testing of ADCC and ASD. Prior to this study, they underwent mutation screening of the coding regions of the *FOXC1* and *PITX2* genes by Sanger sequencing and multiplex-ligation dependent probe amplification (MLPA), as described [27]. A total of four Belgian families and one Belgo-Romanian family were investigated for coding mutations in the *PITX3* gene. Ophthalmological examinations included visual acuity measurements, slit-lamp biomicroscopy, tonometry and fundoscopy. The study was performed in accordance with the Declaration of Helsinki.

### Molecular genetic study of *PITX3*

The coding region of *PITX3* was screened using PCR amplification and subsequent Sanger sequencing. Primer pairs for the coding exons and PCR conditions can be found in Additional file 1. Sequencing was performed with the BigDye Terminator v3.1 Cycle Sequencing Kit on an ABI 3730XL genetic Analyzer, according to the manufacturer’s instructions (Applied Biosystems, Carlsbad, CA). Mutation nomenclature is based on reference transcript NM_005029.3, with +1 corresponding to the A of the translation initiation codon ATG in the cDNA nomenclature, according to the Human Genome Variation Society (HGVS) nomenclature guidelines.

### Functional characterization of *PITX3* mutations

#### DNA constructs, cell culture and luciferase assays

The coding sequences corresponding to human PITX3 wild-type, the previously reported PITX3 p.(Gly220Profs*95) mutant [28] and the novel PITX3 p.(Ser192Alafs*117) mutant were cloned into the modified pcDNA3.1 expression vector (Invitrogen, Carlsbad, CA); all constructs were verified by DNA sequencing. The vector modification included an insertion of a C-terminal FLAG-tag followed by stop codon in frame with the PITX3 wild-type or mutant sequences. The *PITX3* c.573del mutation was generated by QuickChange Lightning Site-Directed Mutagenesis kit (Stratagene, La Jolla, CA) according to the manufacturer’s protocol.

Human lens epithelial cells (B3) (ATCC, Manassas, VA) were cultured in DMEM medium supplemented with 20% fetal calf serum (FBS), glutamine, sodium pyruvate (1 mM) and non-essential amino acids. The luciferase assays were performed with two different luciferase reporter vectors, *bcd*-TK-luc [28] and *MIP656-bcd1,2*-pGL3 [29] and as previously described. The experiments were performed two times in triplicate for both reporters. Student’s paired t-test with a one-tailed distribution was utilized to determine the statistical significance of any differences in activity level.

#### Immunocytochemistry

B3 cells were cultured on coverslips to 50 to 80% confluency, fixed with 4% formaldehyde in PBS and then permeabilized with 0.25% Triton X-100. Indirect immunofluorescent staining was performed with monoclonal anti-FLAG M2 antibody (Sigma, St. Louis, MO) and Alexa Fluor 568 donkey anti-mouse IgG (Invitrogen) as a secondary antibody. Hoechst 33342 (Invitrogen) was used as a nuclear counterstain.

#### Electrophoretic mobility shift assay (EMSA)

Nuclear extracts were prepared from B3 cells transiently transfected with the corresponding plasmids using the CelLytic NuCLEAR extraction kit (Sigma) as previously described [29]. In short, cells were harvested 48 hours after transfection and the cellular pellet was resuspended in hypotonic lysis buffer with protease inhibitor cocktail (Sigma). Igepal CA-630 was added after 15 minutes of incubation on ice, and then the cells were vortexed and spun down. Nuclear proteins were released by incubation of the crude nuclear pellet in the high-salt extraction buffer in the presence of protease inhibitors for 30 minutes on ice followed by centrifugation. Equal concentration of recombinant proteins was verified by Western blot densitometry (ImageJ, NIH, Bethesda, MD). Oligonucleotide, 5′GATCCTAATCCCGTCGCGTCGTAATCCGGATC3′, containing two bicoid sites separated by 10 nucleotides (*Bcd* probe) was used in this study [28]. For oligonucleotide labeling and detection, Biotin 3′ End DNA Labeling Kit (Pierce, Rockford, IL) and LightShift Chemiluminescent EMSA Kit (Pierce) were used in accordance with the manufacturer’s protocol. 20 fmol of biotin end-labeled probe were mixed with binding buffer containing 50 ng/μl of Poly(dI-dC) and 2 μl of nuclear extracts. After 20 minutes of incubation at room temperature, reactions were either diluted with 5× Loading buffer or further incubated for 30 minutes in presence of 1 μg of goat polyclonal Pitx3 N-20 antibody (Santa Cruz Biotechnology, Dallas, TX) for the Supershift assay. Binding reactions and free probe were run on 5% native polyacrylamide gel in 0.5× TBE buffer.

#### Western blot

For Western blot, equal volumes of whole cell extract from transfected cells were electrophoresed into 10% SDS-polyacrylamide gel, transferred to polyvinylidine difluoride filters (Millipore, Billerica, MA) and probed with FLAG-M2 antibodies. After reaction with a secondary antibody conjugated with HRP, signal was detected with ECL Western Blotting Substrate (GE Healthcare, Little Chalfont, UK).

## Results

### *PITX3* mutations and associated phenotypes

#### Recurrent PITX3 mutation

**Figure 1:**
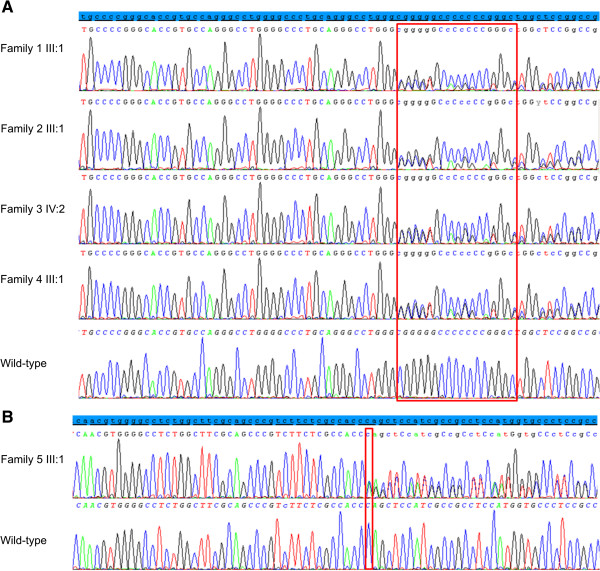
**Sequence electropherograms of the identified *PITX3 *mutations. A.** Sequence electropherograms of the four index patients with the recurrent c.640_656dup *PITX3* mutation. **B.** Sequence electropherogram of the proband with the novel *PITX3* mutation, c.573del. The reference sequence is displayed in blue on the top of each panel. Sequence electropherograms of the wild-type are shown at the bottom of each panel. The mutations are indicated with a red box.

**Figure 2:**
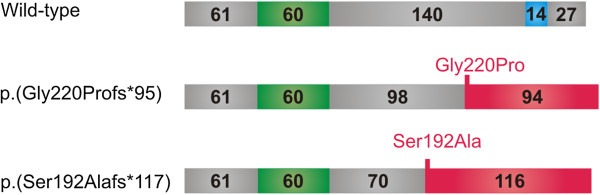
**Schematic representation of mutated PITX3 proteins.** The top diagram represents the wild-type PITX3 protein. The green box represents the homeodomain of 60 amino acids and the blue box the OAR (named after *otp, aristaless* and *rax*) domain of 14 amino acids. The middle and bottom diagram represent the mutant PITX3 proteins p.(Gly220Profs*95) (recurrent) and p.(Ser192Alafs*117) (novel), respectively. The positions of the mutations are indicated with a red line and the resulting aberrant protein segments are highlighted by a red box.

**Figure 3:**
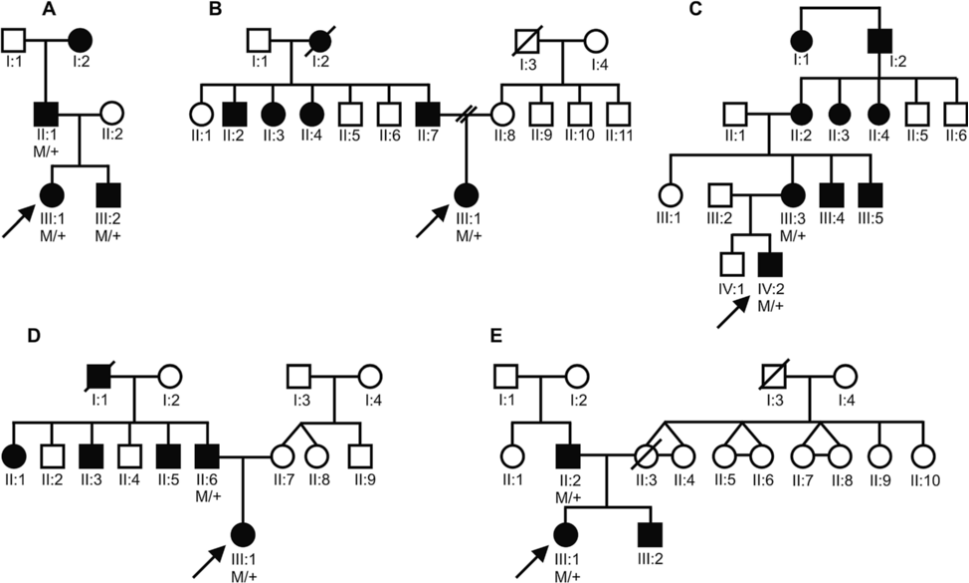
**Pedigrees of Families 1 (A), 2 (B), 3 (C), 4 (D) and 5 (E).** Affected individuals are indicated by filled symbols and index patients by an arrow. In the genotype M stands for the mutant allele (c.640_656dup in Families 1–4 and c.573del in Family 5) and + for the wild-type allele.

In the index patients of four Belgian families, the recurrent duplication of 17-bp, c.640_656dup was found (Figure 1). This duplication creates a frameshift mutation, p.(Gly220Profs*95), leading to the replacement of the normal 82 C-terminal amino acids with 94 erroneous residues (Figure 2). Targeted mutation analysis in available family members demonstrated co-segregation of this mutation with disease (Figure 3).

The first Belgian family is a three-generation family with four affected individuals diagnosed with posterior subcapsular cataract (PSC) (Figure 3A). The recurrent duplication was found in the index patient (III:1), her brother (III:2) and her father (I:1).

**Figure 4:**
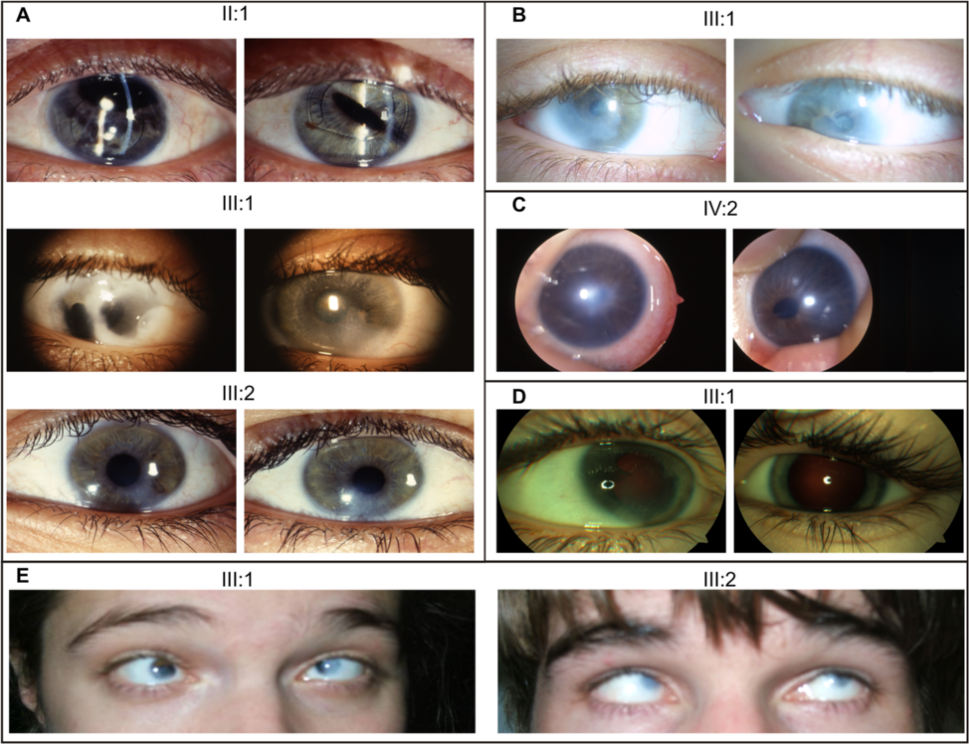
**Clinical pictures.** In this figure, anterior segment pictures are presented for both right (RE) and left eyes (LE). All eyes shown are pseudophakic, except for family 4 (panel **D**). **A.** Both eyes of the father (Family 1, II:1) show corneas with small diameter (10 mm) and normal transparency. An anterior chamber lens implant is observed with iris atrophy and iatrogenic correctopia. The index patient (III:1) underwent a rotational corneal autograft in the RE. Corneal clouding, iridocorneal adhesions and iris atrophy can be seen in the LE. Both eyes of the brother (III:2) show inferior corneal clouding with endothelial haze. **B.** In both eyes of the index patient (Family 2, III:1) corneal opacities can be observed. **C.** In the RE of the index patient (Family 3, IV:2) optical iridectomy was performed because of severe central corneal clouding. In the LE corectopia and corneal haze is observed. **D.** Iridocorneal adhesions and iris hypoplasia are observed in both eyes of the index patient (Family 4, III:1) although more pronounced in the RE. **E.** In the index patient (Family 5, III:1) and her brother (III:2) microcornea (6 mm) and severe corneal opacities are observed.

The index patient (III:1) was born with severe corneal clouding in the right eye for which rotational corneal autografting was performed to obtain a clear optical axis, however this eye remained without functional visual acuity (VA). When she was 13 years old, she underwent lens extraction with lens implant of her left eye. Later on, she developed severe corneal endothelial cell loss in this eye and therefore underwent corneal transplant at the age of 15. A follow-up ophthalmological examination at the age of 26 revealed a VA for her right and left eye of light perception and 0.1, respectively, and corneal clouding with significant corneal endothelial loss. Her brother (III:2) also had congenital cataract with ASD. He was pseudophakic with a significant loss of endothelial cells. VA for his right and left eye was 0.5 and 0.6, respectively. Their father (II:1) has isolated bilateral congenital cataract. He underwent cataract extraction at the age of 18. No other corneal or anterior segment anomalies were observed in him (Figure 4). All affected individuals (III:1, III:2 and II:2) have normal intraocular pressure (IOP).

In the second Belgian family there are three generations of affected individuals with ADCC (Figure 3B). The recurrent mutation c.640_656dup was found in the index case III:1; no DNA from other family members was available for targeted mutation testing. The cataracts of the index patient (III:1), her father (II:7), three paternal uncles (II:2, II:3, and II:4), and her paternal grandmother (I:2) developed gradually during childhood and became clinically apparent at different ages necessitating cataract extractions. Bilateral cataract extraction was performed in the index patient (III:1) at the age of 7 resulting in a bilateral best corrected visual acuity (BCVA) of 0.2. Apart from the cataract, the index patient has microcornea (bilateral corneal diameters of 9 mm; microcornea is defined as a corneal diameter < 10 mm [30]), corneal opacities, iridocorneal adhesions, glaucoma and nystagmus (Figure 4). Over a period of three years she was admitted twice for retinal detachment in her left eye. In addition, the index patient has intellectual disability. But as her mother and one maternal uncle (II:11) also display intellectual disability, this was felt to be unrelated to the paternally inherited *PITX3* mutation.

The third Belgian family is a four-generation PSC family with nine affected individuals (Figure 3C). The recurrent duplication was found in the index patient (IV:2) and his mother (III:3). The index patient was diagnosed with bilateral congenital cataract and ASD. As severe central corneal clouding was present in his right eye, he underwent optical iridectomy however this eye remained amblyopic. Cataract extraction of his left eye was performed at the age of 8. After the extraction, the patient’s VA improved from 0.4 to 0.7 for his left eye while the VA for his right eye was light perception (Figure 4). His mother (III:3) presented only with bilateral congenital cataracts that were extracted at the age of 6. Her affected brothers (III:4 and III:5) and mother (II:2) underwent cataract extraction at the age of 17, 10 and 7 respectively.

The fourth Belgian family encompasses three generations of affected individuals with ADCC (Figure 3D). The recurrent duplication was found in the index case III:1; no DNA from other family members was available for targeted mutation testing. The index patient (III:1) is 3.5 years old and presented with nystagmus (manifesting latent left eye), corneal haze and iridocorneal adhesions with endothelial loss. The developmental anomaly was more severe in the right eye. VA for her right and left eye is light perception and 0.4, respectively. Her IOP was 12 mmHg (Figure 4). Her father (II:6) only presented with congenital cataract that was extracted at the age of 12.

#### Novel PITX3 mutation

In a Belgo-Romanian family with ADCC a novel 1-bp deletion c.573del was found in exon 4, p.(Ser192Alafs*117), in the index patient (III:1) (Figure 1). Targeted testing identified this 1 bp-deletion in her father (II:2) as well; no DNA samples were available from other family members (Figure 3E). This 1-bp deletion creates a frameshift starting at codon 192 and introduces 116 novel residues before resulting in a stop codon downstream of the original stop codon (Figure 2). The index patient (III:1) was diagnosed with congenital cataract for which she underwent cataract extraction when she was 1 year old. Follow-up ophthalmological examination at the age of 25 demonstrated a VA for her right and left eye of light perception and 0.02, respectively, bilateral microcornea (6 mm), corneal opacities and iridocorneal adhesions. She underwent optical iridectomy of both eyes. Her IOP was normal. Her brother (III:2) has microcornea (5 mm) and corneal opacities in addition to congenital cataract (Figure 4). Their father (II:2) has microcornea and congenital cataract.

### Functional characterization of novel *PITX3* mutation p.(Ser192Alafs*117) compared to the recurrent mutation p.(Gly220Profs*95)

#### Subcellular localization

**Figure 5:**
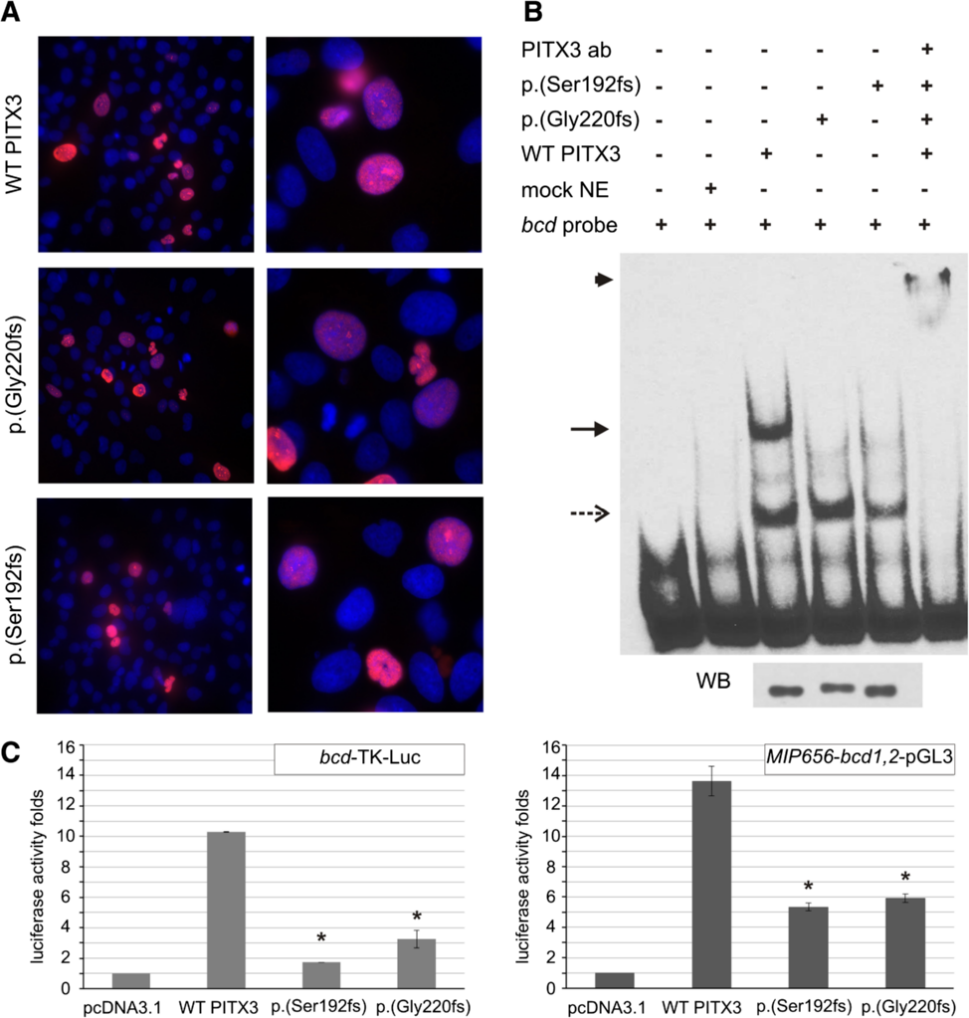
**Subcellular localization (A), DNA-binding (B) and transactivation activity (C) of the novel *PITX3 *mutation in comparison with the recurrent mutation. A.** Immunocytochemistry of the PITX3 wild-type and mutant proteins transfected in B3 lens epithelial cells. Cells were stained with monoclonal anti-FLAG M2 primary antibody and Alexa Fluor 568 donkey anti-mouse IgG as a secondary antibody (red); Hoechst 33342 was used as a nuclear counterstain (blue). All three proteins localize predominantly to the nucleus. **B.** Results of EMSA experiments demonstrating the DNA-binding capacity of the PITX3 wild-type and mutant proteins. The profile of the wild-type PITX3 protein (third from the left) displays two major bands likely representing DNA interactions with PITX3 homo- or hetero-dimers (upper band; arrow) or PITX3 monomers (lower band; dashed arrow). The profile of the mutant PITX3 proteins only shows the lower band. Addition of anti-PITX3 antibody results in the appearance of a supershifted band (asterisk) and disappearance of the other bands. Western blot (bottom) shows similar levels of PITX3 wild-type and mutant proteins in the nuclear extracts used for EMSA experiments. **C.** Luciferase assay results for wild-type and mutant PITX3 co-transfected with the *bcd*-TK-luc or *MIP656*-*bcd1,2*-pGL3 reporters in human lens epithelial cells. The values are reported as fold changes of luciferase activity in comparison to an empty vector (pcDNA3.1). All luciferase activities were normalized to β-galactosidase activity and error bars indicate the standard deviation of two independent experiments performed in triplicate. The asterisk indicates the statistically significant differences in fold change (p < 0.001).

Consistent with its role as a transcription factor, PITX3 was found to localize to the nucleus [28]. The novel PITX3 mutant p.(Ser192Alafs*117) displayed a similar nuclear localization as observed for the wild-type PITX3 and the previously reported recurrent mutant p.(Gly220Profs*95) (Figure 5A).

#### Assessment of DNA-binding

It has been previously shown that *PITX3* mutations result in decreased DNA-binding activities [28]. To assess if the novel p.(Ser192Alafs*117) mutation has a similar effect on DNA-binding activity, we performed EMSA for the novel mutant in comparison to the previously reported recurrent p.(Gly220Profs*95) mutant and wild-type proteins. The results were also compared to the EMSA profiles of the *bcd* probe alone and with mock nuclear extracts (NE) to exclude nonspecific binding activity. The observed profile for the wild-type protein consisted of two major bands in agreement with previously reported results (Figure 5B). The fast-migrating band probably corresponds to PITX3 binding DNA as a monomer and the slower migrating bands may represent PITX3 homodimer- or PITX3 heterodimer-DNA complexes with other proteins [13,28]. The slower migrating bands have disappeared in the EMSA profiles of both PITX3 mutants while the fast-migrating band, likely representing monomeric DNA-binding activity, remained. The specificity of the assay was verified by adding an anti-PITX3 antibody resulting in a supershifted band and fading of the other bands (Figure 5B). Western blot analyses were performed to confirm the equal presence of wild-type and mutant PITX3 protein in the nuclear extract used for the EMSA experiments (Figure 5B).

#### Transactivation activity

In addition to altered DNA-binding properties, it has also been shown that PITX3 mutants exhibit a reduced transactivation activity [28]. Using luciferase assays in human lens epithelial cells we evaluated the transactivation activity of the novel PITX3 mutant p.(Ser192Alafs*117) in comparison to the recurrent p.(Gly220Profs*95) mutant and wild-type proteins (Figure 5C). Co-transfection of the wild-type PITX3 with the *bcd*-TK-luc reporter resulted in a ~10-fold increase in luciferase activity in comparison with an empty pcDNA3.1 vector, while the novel p.(Ser192Alafs*117) and recurrent p.(Gly220Profs*95) mutants resulted in a ~1.7-fold (16.8% of wild-type activity; p < 0.001) and ~3.2 fold increase (31.5% of wild-type activity; p < 0.001), respectively. A similar trend could be observed using the *MIP656*-*bcd1,2*-luc reporter, as a ~13-fold increase in luciferase activity was detected when this reporter was co-transfected with wild-type PITX3. In contrast, the luciferase activity for the novel and recurrent PITX3 mutants was found to be 39.2% and 43.4% (p < 0.001) of wild-type activity, respectively.

## Discussion

Congenital cataract is a clinically and genetically heterogeneous condition. Most genes are associated with isolated congenital cataracts while mutations in the transcription factor genes *PAX6*, *FOXE3*, *EYA1*, *MAF*, and *PITX3* lead to congenital cataract with ASD [3-7]. Of particular note, *PITX3* mutations can cause isolated congenital cataract as well as ASD-associated cataract, even within the same family [7]. This inter- and intrafamilial phenotypic variability can likely be ascribed to modifier genes or environmental factors. *PITX3* mutations represent a rare cause of congenital cataract with or without ASD. Indeed, only five unique *PITX3* mutations in 13 different families were known before the onset of this study (Table 1). The most common mutation is the 17-bp duplication c.640_656dup accounting for more than half of the families [7,18-21,23,24] and ADCC with ASD has been documented only in association with this duplication. Notably, the novel *PITX3* mutation c.573del, p.(Ser192Alafs*117), was found in a family with ADCC and ASD, thus being the second mutation leading to this phenotype. Like the other reported *PITX3* mutations, this novel frameshift mutation is located outside the homeodomain. Interestingly, all *PITX3* frameshift mutants lack the C-terminal 14-amino-acid motif (see Additional file 2), which is conserved in numerous paired-like homeodomain proteins and is named the OAR domain, after the homeodomain genes *otp*, *aristaless*, and *rax*[14]. The function of this OAR domain is not fully established yet but it has been suggested that it could interact with additional proteins, hence mediating the target specificity of the homeobox-containing transcription factors [13,14]. Although the OAR domain is conserved in all of these homeodomain proteins, its function appears to vary among the proteins. Functional assays with different deletion mutants of Otp showed that mutants missing the region downstream of the homeodomain, including the OAR domain, presented with a different DNA-binding profile and a substantial reduction of transactivation activity, thus suggesting an activating regulatory role [31]. The OAR domain is also important for the transactivation activity of SHOX as a truncated SHOX mutant lacking this domain has an abolished transactivation activity [32]. In contrast, a study of the Pitx2 C-terminus demonstrates an inhibitory role for the OAR domain as it was shown that the presence of this domain inhibits DNA-binding while masking this domain through protein interaction stimulates DNA-binding and transcriptional activation [33]. This finding was supported by studies in Prx2 and Cart1 where the OAR domain inhibits transactivation and serves as an intramolecular switch of its transactivation activity, respectively [34,35]. To explore the function of the C-terminal region in PITX3*,* Sakazume et al. investigated the effect of PITX3 mutants on DNA-binding and transactivation activity. In accordance with the Otp and SHOX study, an altered DNA-binding profile and diminished transcriptional activity was shown [28].

**Table 1:** **Overview of the different *PITX3 *** **mutations described in human and associated phenotypic details**

**Mutation**	**Mode of inheritance**	**Clinical features**	**Reference**
c.38G > A, p.(Ser13Asn)	Dominant	Mother and son with ADCC and glaucoma at a later age.	7
c.542del, p.(Pro181Leufs*128)	Dominant	Four-generation English family with isolated PPC.	26
c.573del, p.(Ser192Alafs*117)	Dominant	Belgo-Romanian family with ADCC. Two individuals also have ASD.	This study, Family 5
c.640_656dup, p.(Gly220Profs*95)	Dominant	Six-generation family with ADCC and ASD.	7
		Four-generation English family with PPC. One individual also has ASD and congenital glaucoma.	18, 19
		Four-generation English family with PPC. Four individuals also have ASD.	18, 19
		Five-generation English family with isolated PPC.	18, 19
		Four-generation Chinese family with isolated PPC.	18
		Four-generation British/German family with isolated PPC.	20
		Five-generation English family with isolated PPC.	21
		Four-generation Australian family with PSC. Seven individuals also have ASD.	23, 24
		Three-generation Belgian family with PSC. Two individuals also have ASD.	This study, Family 1
		Three-generation Belgian family with ADCC. One individual also has ASD.	This study, Family 2
		Four-generation Belgian family with PSC. One individual also has ASD.	This study, Family 3
		Three-generation Belgian family with ADCC. One individual also has ASD.	This study, Family 4
c.640_656del, p.(Ala214Argfs*42)	Recessive	Daughter from healthy first cousin parents with ASD and severe congenital microphthalmia.	25
c.650del, p.(Gly217Alafs*92)	Dominant	Four-generation Hispanic family with isolated cataract.	18
	Dominant/recessive	Three-generation Lebanese family with PPC. Two brothers from a consanguineous mating showed a more severe ocular and neurologic phenotype in addition to PPC.	22
Microdeletion of 10q24.32 encompassing *PITX3*	*De novo*	Patient with Smith–Magenis syndrome-like behavioural abnormalities, intellectual disability and dysmorphic features but no eye phenotype.	41

Mutation nomenclature is based on reference transcript NM_005029.3 and the HGVS guidelines.

ADCC: autosomal dominant congenital cataract; PPC: posterior polar cataract; ASD: anterior segment dysgenesis; PSC: posterior subcapsular cataract.

Both the novel and recurrent *PITX3* mutations found here disrupt the C-terminus including the OAR domain. As they both lead to a similar phenotype, we assessed whether this similarity could be substantiated by *in vitro* functional assays. Since all *PITX3* frameshift mutations utilize a stop codon located downstream of the normal stop codon in the last coding exon, they are not predicted to be subject to nonsense mediated decay (NMD). The novel PITX3 mutant retained its nuclear localization but displayed altered DNA-binding properties similar to the recurrent PITX3 p.(Gly220Profs*95) mutant. In addition, a similar decrease of transcriptional activity compared to wild-type PITX3 was observed for the novel and recurrent PITX3 mutants. These mutations may produce aberrant protein that can either antagonize DNA-binding activity of wild-type proteins or form inactive dimers with wild-type proteins, impairing wild-type protein function in both situations. Co-transfection assays with mutant and wild-type PITX3 could not, however, substantiate a dominant-negative effect [28]. A loss-of-function effect for the *PITX3* mutations is therefore more probable. This view is supported by the report on a large ADCC pedigree in which two brothers from a consanguineous mating are homozygous for the *PITX3* mutation segregating in the family and manifest a more severe ocular and neurological phenotype, with severe microphthalmia and neurological involvement in addition to cataract [22]. This severe combination of ocular and neurological involvement has also been described in the two spontaneous *Pitx3* mutant mice, *aphakia* and *eyeless*[9,10,15,36]. Interestingly, besides its expression in the developing lens, *Pitx3* expression was also observed in the dopamine neurons of the midbrain [10]. A severe loss of dopaminergic neurons in the substantia nigra was also observed in *aphakia* and *eyeless* mice. As these neurons play important roles in the control of movement, emotion, cognition, and reward related behavior, this neuronal loss can explain the observed locomotor and behavioural defects [36-39]. Unlike the human *PITX3* mutations, heterozygous *Pitx3* mice were phenotypically normal which might be attributed to interspecies differences, as mice seem to be less sensitive to gene dosage. However, the loss-of-function hypothesis is challenged by the report on another consanguineous mating between phenotypically normal first cousins resulted in a girl homozygous for the *PITX3* mutation c.640_656del, manifesting as severe bilateral microphthalmia and ASD but without cataract or a neurological phenotype [25]. This phenotype is reminiscent of the manifestations in the Texel Sheep as this breed displays microphthalmia as an autosomal recessive congenital condition, caused by a missense mutation in the conserved homeodomain of *PITX3*, c.338G > C, p.(Arg113Pro) [40]. In addition, a heterozygous microdeletion of 10q24.32 encompassing *PITX3* in a patient with Smith–Magenis syndrome-like behavioral abnormalities was recently described. The patient also presented with intellectual disability and dysmorphic features but, surprisingly, lacked an eye phenotype. Analysis of neurotransmitters in his cerebrospinal fluid revealed an absence of L-DOPA and L-DOPA treatment led to mild improvement of his behavior [41]. Based on these observations and the selective loss of dopaminergic neurons in the *Pitx3* mutant mice, it might be interesting to assess the dopaminergic function of patients with *PITX3* mutations.

Taken together, these findings suggest that *PITX3* associated phenotypes most likely result from a more complex mutational mechanisms than loss of function effects alone and that more extensive studies are needed to correlate the phenotypic and molecular consequences of *PITX3* mutations.

## Conclusions

In this study, we identified a novel *PITX3* mutation, p.(Ser192Alafs*117) and the recurrent p.(Gly220Profs*95) mutation in one Belgo-Romanian family and four Belgian families with ADCC and ASD, respectively. The novel mutation p.(Ser192Alafs*117) is only the second *PITX3* mutation to be associated with this combined phenotype, along with the recurrent mutation p.(Gly220Profs*95). The similarity in phenotypic expression could be explained by *in vitro* functional studies which demonstrated similar molecular consequences for the novel p.(Ser192Alafs*117) and the recurrent p.(Gly220Profs*95) mutations.

## Competing interests

The authors declare that they have no competing interests.

## Authors’ contributions

HV carried out the molecular genetic studies and drafted the manuscript. EAS performed the immunocytochemistry, EMSA experiments and luciferase assays. FM, TDR and IC performed the ophthalmological examinations and provided clinical information. EVS and EDB participated in the design and coordination of the study, and helped to draft the manuscript. All authors read and approved the final manuscript.

## Supplementary Material

Additional file 1 **PCR primers and conditions for molecular screening of *PITX3*.** Click here for file

Additional file 2 **Schematic representation of all reported mutated PITX3 proteins.** The top diagram represents the wild-type PITX3 protein. The green box displays the homeodomain of 60 amino acids and the OAR (named after *otp*, *aristaless* and *rax*) domain of 14 amino acids is displayed by a blue box. The recurrent p.(Gly220Profs*95) and novel p.(Ser192Alafs*117) mutations are indicated in bold. The positions of the mutations are indicated with a red line and a red box displays the resulting aberrant protein segments. Click here for file
