# Supplementary material for: Novel and recurrent PITX3 mutations in Belgian families with autosomal dominant congenital cataract and anterior segment dysgenesis have similar phenotypic and functional characteristics
Source: Orphanet J Rare Dis. 2014 Feb 20;9:26. doi: 10.1186/1750-1172-9-26 (PMC3937428; doi:10.1186/1750-1172-9-26)
Supplement: Additional file 1 — PCR primers and conditions for molecular screening of PITX3 . [file 1750-1172-9-26-S1.pdf]

Additonal file 1

PCR primers:

Exon	Forward primer (5' – 3')	Reverse primer (5' – 3')	Amplicon length
2	acgcagccccagctttac	aagccagcgcatattctcc	395
3	gtgcaggacataacagcttc	gagcagaggctggaggttg	528
4	ccgtctctagccacctcatc	gaccgagttgaaggcgaat	338
4	agacctttccattcgccttc	agtcaaaatgaccccagtcc	483

PCR conditions:

- 100 ng DNA
- 1× PCRx Amplification Buffer (Invitrogen, Carlsbad, CA)
- 2× PCRx Enhancer Solution (Invitrogen)
- 0.2 mmol/l of each dNTP (Invitrogen)
- 1.5 mmol/l $MgSO_4$ (Invitrogen)
- 1 unit PlatinumTaq DNA polymerase (Invitrogen)
- 0,50 µmol/l of each oligodeoxynucleotide primer (IDT, Coralville, IA)

Total volume: 15 µl

Cycling conditions:

<table>
<tr><th>PCR step</th><th>Temperature</th><th>Time</th></tr>
<tr><td>Initial denaturation</td><td>94 °C</td><td>5 min</td></tr>
<tr><td rowspan="3">10 touchdown cycles (annealing temperature decreases 1 °C each cycle)</td><td>94 °C</td><td>30 sec</td></tr>
<tr><td>65 °C</td><td>15 sec</td></tr>
<tr><td>72 °C</td><td>60 sec</td></tr>
<tr><td rowspan="3">35 amplification cycles</td><td>94 °C</td><td>45 sec</td></tr>
<tr><td>55 °C</td><td>45 sec</td></tr>
<tr><td>72 °C</td><td>90 sec</td></tr>
<tr><td>Final extension</td><td>72 °C</td><td>10 min</td></tr>
</table>
