# Supplementary material for: Novel and recurrent PITX3 mutations in Belgian families with autosomal dominant congenital cataract and anterior segment dysgenesis have similar phenotypic and functional characteristics
Source: Orphanet J Rare Dis. 2014 Feb 20;9:26. doi: 10.1186/1750-1172-9-26 (PMC3937428; doi:10.1186/1750-1172-9-26)
Supplement: Additional file 2 — Schematic representation of all reported mutated PITX3 proteins. The top diagram represents the wild-type PITX3 protein. The green box displays the homeodomain of 60 amino acids and the OAR (named after otp, aristaless and rax) domain of 14 amino acids is displayed by a blue box. The recurrent p.(Gly220Profs*95) and novel p.(Ser192Alafs*117) mutations are indicated in bold. The positions of the mutations are indicated with a red line and a red box displays the resulting aberrant protein segments. [file 1750-1172-9-26-S2.pdf]

Additional file 2

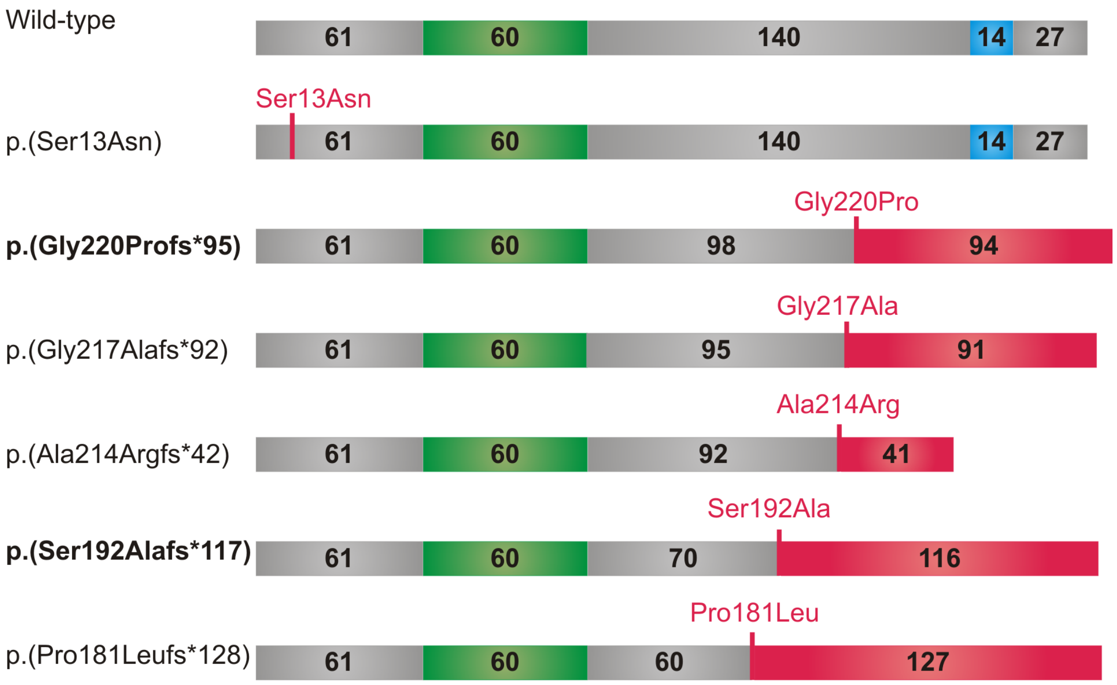

Additional Figure 1. Schematic representation of all reported mutated PITX3 proteins

The top diagram represents the wild-type PITX3 protein. The green box displays the homeodomain of 60 amino acids and the OAR (named after *otp*, *aristaless* and *rax*) domain of 14 amino acids is displayed by a blue box. The recurrent p.(Gly220Profs*95) and novel p.(Ser192Alafs*117) mutations are indicated in bold. The positions of the mutations are indicated with a red line and a red box displays the resulting aberrant protein segments.
